# Variations in burn perfusion over time as measured by portable ICG fluorescence: A case series

**DOI:** 10.4103/2321-3868.142397

**Published:** 2014-10-25

**Authors:** Sharmila Dissanaike, Senan Abdul-Hamed, John A. Griswold

**Affiliations:** Department of Surgery, Texas Tech University Health Sciences Center, Mailstop 8312, 3601 4th St, Lubbock, Texas 79430 USA

**Keywords:** Burn depth assessment, indocyanine green, SPY

## Abstract

The early determination of healing potential in indeterminate thickness burns may be difficult to establish by visual inspection alone, even for experienced burn practitioners. This case series explores the use of indocyanine green (ICG) fluorescence using portable bedside assessment as a potential tool for early determination of burn depth. Three subjects with indeterminate thickness burns had daily perfusion assessment using ICG fluorescence assessment using the SPY machine (SPY®, Lifecell Corp., NJ, USA) in addition to standard burn care. The fluorescence was quantified as a percentage of the perfusion of intact skin, and areas of hypo- and hyper-perfusion were indicated. The study was concluded when the burn surgeon, blinded to the ICG results, made a clinical determination of the need for skin grafting or discharge. The perfusion in areas of differing depth of burn were compared over the entire study period to determine both the magnitude of difference, and the point in the time course of healing when these changes became evident. Significant differences in perfusion were noted between burned areas of varying depth. These differences were evident as early as the first post-burn day, and persisted till the completion of the study. ICG fluorescence represents a potential adjunct in burn assessment in this first longitudinal study of its use; however much more systematic research will be required to judge the feasibility of clinical implementation.

## Introduction

While it is relatively easy for experienced burn practitioners to differentiate between burns that are almost certainly going to heal (first degree and superficial second degree burns) and those that will almost certainly not heal and instead require excision and skin grafting (deep second and third degree burns), the quest to determine the healing potential of indeterminate burns has occupied burn specialists for decades. The early recognition of burns that will not heal without scarring allows for earlier definitive surgical therapy — excision and split thickness skin grafting, reducing hospital length of stay as well as improving the final cosmetic and functional result, compared to delayed surgery. In attempts to achieve this outcome, some centers advocate a policy of early operation on most second degree burns; however this approach necessarily subjects some patients to unnecessary surgery. A common option is a wait-and-see approach, with daily or otherwise frequent evaluation of the burn by the senior burn surgeon, with visual assessment of likelihood of healing. This process may involve 5–7 days of daily assessment, and thus may result in a delay to definitive surgery in some patients, and a potentially unnecessary prolongation of hospital stay in others. Alternatives to visual inspection have been proposed by many authors, and of these, laser Doppler assessment has received the most attention.[1–4]

**Table 1:** 

Access this article online	
**Quick Response Code**:	**Website**: www.burnstrauma.com
	**DOI**: 10.4103/2321-3868.142397

The use of indocyanine green (ICG) fluorescence was first described by Sheridan *et al.*,[5] in a groundbreaking trial of 10 patients based on successful preliminary results in a porcine model. They showed clear differences in perfusion in deep burns compared to superficial burns, thus establishing proof of principle. Still *et al.*, subsequently corroborated these results with a trial in nine patients in 2001.[6] A recent study using an animal model also confirmed the potential for ICG to serve as a tool in assessing burn injury.[7] However both clinical studies only used the imaging technique at a single timepoint after burn, and due to limitations of the technology at that time, could only perform visual assessment of the fluorescein images. In addition, the equipment required to perform the study meant that this was usually a cumbersome process, requiring the patient to be moved into a room with specialized heavy equipment similar to an angiography suite.

The assessment of ICG fluorescence has now been incorporated into a portable commercial device (SPY®, Lifecell Corp., NJ, USA) that is in widespread clinical use for assessing the perfusion of intestinal anastomosis and myofasciocutaneous flaps, thus significantly reducing the logistical difficulty of performing the study. Although previous researchers have shown that ICG fluorescence might be helpful in burn depth assessment, their use of only one measurement at an arbitrary time point from injury significantly limits the generalizability of their findings. Therefore we performed a pilot study using ICG fluorescence to map the areas of an indeterminate burn on a daily basis during the course of initial hospitalization, in order to evaluate the difference in baseline perfusion as well as perfusion changes in areas of different burn depth. In addition, we used the currently available improved technology to calculate perfusion as a percentage of the perfusion of intact skin, thus providing a much more precise estimate of perfusion than was previously possible.

The goal of this study was to identify perfusion differences between areas of different depth, and to determine how early in the post-burn time course these differences became apparent, thus evaluating the potential of portable ICG perfusion measurement to determine likelihood of healing in burns.

## Methods

This study was approved by the Texas Tech University Health Sciences Center Institutional Review Board.

### Study conduct

Patients with burns of indeterminate thickness and less than 15% TBSA, as determined by an attending burn surgeon on admission, were screened for inclusion in the study. After informed consent, each subject had daily dressing changes and wound care per our standard burn center protocol. In addition, SPY assessment was performed during every daily dressing change. This involved an intravenous dose of 5 mg of ICG being injected, followed by fluorescence images being captured of the burn. Each patient received the same 5 mg dose which was not weight-adjusted, as per FDA approved protocol when the SPY device is used for other indications. While repeat doses are routinely used in clinical practice and doses up to 50 mg at one setting have been documented as safe, the lowest effective dose was chosen in order to ensure patient safety for the purposes of this study.

The SPY machine was activated and the area to be assessed placed under the viewer. Digital photographs of the area were captured using the built-in camera on the SPY machine. Once the ICG was injected, images of perfusion began appearing on screen within 1–2 min, and image capture was begun as soon as this occurred. Image capture continued until the ICG began to washout, a process that usually is complete at around 5 min. Quantification of perfusion was performed on the saved images as described below. This process was repeated daily until a determination of need for skin grafting or readiness for discharge was made. The attending burn surgeon was blinded to the results of the SPY assessment until the study was completed, and thus all clinical decisions were made based on standard visual assessment as per usual protocol.

### Quantification of perfusion

The SPY machine calculates the perfusion in a given area as a percentage based on a reference area indicated by the operator. Intact, unburned skin was selected as the reference for measurement of perfusion, and was therefore set to 100%. This allowed for areas of both hyperemia and hypoperfusion to be documented relative to intact skin. At the conclusion of the study, the perfusion in an area that was determined to be superficial by visual assessment was compared with that of a deeper area and the difference was quantified using perfusion percentage. Values for these same areas for each day of the study were retrospectively compared as a within-subject comparison with the unburned skin set as the standard.

### Clinical care

Wound care was performed using standard burn center protocol, and consisted of non-adhesive gauze impregnated with either Neosporin® or Sulfamylon® ointment. The burn surgeon, who was blinded to the SPY results, assessed the burn visually each day after the SPY procedure was complete. The SPY results were not used to guide treatment decisions in any way, and the study was terminated when the surgeon determined that the burn either required grafting, or was healing and the patient could be discharged.

## Results

### Case 1

**Figure 1:**
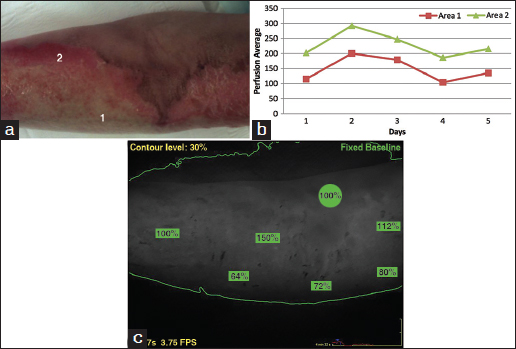
Representative photographs of burn in case 1. (a) Area 1 shows deeper burn and area 2 shows the more superficial burn. (b) Corresponding perfusion graphs showing perfusion in each area over the time course of the study. Intact, unburned skin is set as the reference at 100%. (c) Perfusion images of the areas in photograph. Areas of measured perfusion are shown in green with corresponding percentages. The perfusion scans are from day 3 in case 1.

A 54-year-old female was admitted with 7% TBSA burn to the left arm and hand following a cooking-related flame injury. She underwent daily assessment for 5 days, after which time it was determined that while certain areas of the burn were likely to heal, the majority of the burn required skin graft, and the study was concluded. The perfusion and photographic images are shown in Figure 1.

Area 1 was an area determined to require grafting, while area 2 was judged likely to heal by clinical visual assessment. The difference in perfusion of areas 1 and 2 is evident on the first day post-burn, with area 2 having almost double the perfusion of area 1. This magnitude of difference remained steady throughout the study period, despite the absolute values varying over time. Both areas were excised and grafted at operation to provide uniformity and prevent hypertrophic scarring along the wound edges.

### Case 2

**Figure 2:**
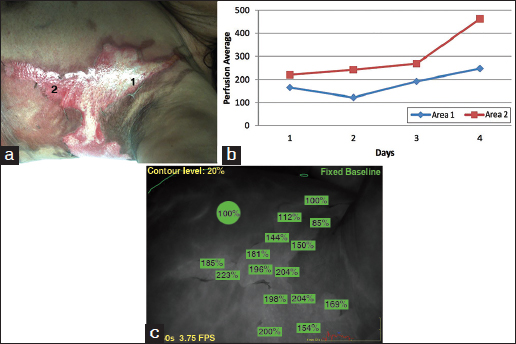
Representative photographs of burn in case 2. (a) Area 1 shows deeper burn and area 2 shows the more superficial burn. (b) Corresponding perfusion graphs showing perfusion in each area over the time course of the study. Intact, unburned skin is set as the reference at 100%. (c) Perfusion images of the areas in photograph. Areas of measured perfusion are shown in green with corresponding percentages. The perfusion scans are from day 3 in case 2.

A 72-year-old female was admitted with 10% TBSA burn to left thigh, left flank and left upper chest also following a cooking-related accident. She was assessed daily for 5 days and was discharged home after we determined that her wounds will heal with no surgery. The perfusion and photographic images are shown in Figure 2.

The difference in area 1 and 2 in this case were smaller than that in the previous case, likely due to a smaller magnitude of difference in burn depth. Unlike Case 1, this patient demonstrated a spike in perfusion on day 4, immediately prior to the assessment of healing and discharge.

### Case 3

**Figure 3:**
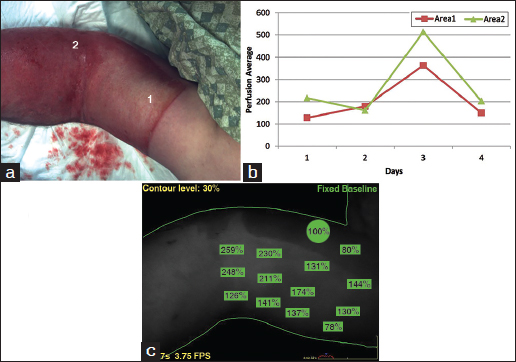
Representative photographs of burn in case 3. (a) Area 1 shows deeper burn and area 2 shows the more superficial burn. (b) Corresponding perfusion graphs showing perfusion in each area over the time course of the study. Intact, unburned skin is set as the reference at 100%. (c) Perfusion images of the areas in photograph. Areas of measured perfusion are shown in green with corresponding percentages. The perfusion scans are from day 3 in case 3.

A 37-year-old male was admitted with 7% TBSA burn to bilateral upper extremities following a motor vehicle accident. His wounds were evaluated for 7 days and the patient was sent home after it was determined that no surgical treatment was indicated. The perfusion and photographic images are shown in Figure 3.

Similar to Case 2, this patient showed a spike in perfusion of both areas on day 3, which then returned to a level similar to previous values on day 4. The absolute difference between areas 1 and 2 was of a smaller magnitude compared to the previous cases, and on day 2 the values were similar enough to be indistinguishable.

Neither of the latter two patients required subsequent skin grafting, suggesting that the correct surgical decision had been made based on clinical grounds alone. In all cases the areas determined to be deeper by visual assessment were shown to have reduced perfusion by quantitative assessment; however as indicated above, the magnitude of difference and absolute values both varied substantially between patients and both areas eventually healed.

## Discussion

This pilot study demonstrates that ICG fluorescence assessment has the potential to differentiate depth of burn as early as day 1, several days prior to this becoming apparent to an expert burn surgeon on visual assessment. It is telling that these changes in perfusion are subtle enough that they are only clearly evident when the computerized system of calculating perfusion as a percentage of baseline is used; the changes in perfusion are difficult to appreciate on visual inspection of the fluorescence images alone. This of course reflects the difficulty burn physicians face using visual assessment — the differences in regions of healing are indeed subtle, and often require technological aids in order to make this determination at an early stage. Early reports of ICG fluorescence demonstrated the technique in burns that were clearly obvious on visual inspection to be either very deep or superficial.[5,6] With computer-aided measurement of perfusion, the technology appears to have evolved to a level that allows detection of subtle differences in healing in indeterminate burns, which increases the potential for clinical utility.

The authors intend this case series to suggest proof-of-concept, rather than make a definitive statement on the clinical potential of this technology in burns. Deeper areas clearly had less perfusion than more superficial areas; however the pattern of variations in perfusion is intriguing. The two patients who did not require skin grafting displayed clear spikes in perfusion immediately before the time that the determination of healing was made — a finding that was not seen in the patient who required skin grafting. The patients that healed also had more variability in the perfusion curves of the deeper and superficial areas, while the first patient who had two clearly distinct areas, one which required grafting and one which would have otherwise healed, showed nearly parallel lines in perfusion. Larger studies will be required to confirm that these findings represent biologic phenomena rather than random variation amplified by the small sample size.

One drawback to this method is the need for an intravenous injection of the ICG which increases the invasiveness of the assessment. ICG is an FDA approved and well-tested drug with indications for perfusion assessment in many other areas, and a long track record of safe use; nevertheless this is a significant drawback compared to completely non-invasive methods. While the generic ICG is available at low cost ($50 per dose from our institutional pharmacy) the proprietary kit that is sold by the SPY company is considerably more expensive ($1500 per hospital contract at this institution), and therefore it likely to be cost-prohibitive for routine daily use. Currently the standard contract between the company and hospital require use of the commercial kit rather than generic ICG, and thus the cost per patient would be significant.

There are several limitations to the generalizability of these results, besides the obvious limitation of a small descriptive case series. Firstly, while the measured perfusion clearly varied with depth of burn, this difference was apparent even when both areas were relatively deep, or relatively superficial i.e. the perfusion difference compared to intact skin seems to be a relatively linear continuous variable rather than an ordinal or exponential variable. As such, it is likely to be difficult to generate a clear cut-off point to make decisions on operative treatment. There was no significant difference in absolute perfusion in the case that required surgery and the cases that healed, which is a warning that clinical applicability might be limited. The absolute value of perfusion varied substantially between cases, suggesting that variability between individuals may also pose a problem with this form of assessment. Finally, there was substantial variability in perfusion between day 3 and 4 in the latter two cases, which indicates that timing of the intervention will significantly affect the results, and would have to be standardized in order to create a clinically useful assessment tool.

## Staying in Touch with the Journal

### (1) Table of Contents (TOC) email alert

Receive an email alert containing the TOC when a new issue of the journal is available online. To register for TOC alerts go to http://www.burnstrauma.com/signup.asp.

### (2) Connect to the journal on social media

Users can contact the editorial office of Burns & Trauma on facebook, LinkedIn and weChat.

## References

[1] Hoeksema H, Baker RD, Holland AJ, Perry T, Jeffery SL, Verbelen J, *et al.* A new, fast LDI for assessment of burns: A multi-centre clinical evaluation. Burns 2014. 10.1016/j.burns.2014.04.024 24996246

[2] Sharma VP, O’Boyle CP, Jeffery SL (2011). Man or machine? The clinimetric properties of laser Doppler imaging in burn depth assessment. J Burn Care Res.

[3] Gill P (2013). The critical evaluation of laser Doppler imaging in determining burn depth. Int J Burns Trauma.

[4] Hop MJ, Hiddingh J, Stekelenburg C, Kuipers HC, Middelkoop E, Nieuwenhuis MK (2013). LDI study group. Cost-effectiveness of laser Doppler imaging in burn care in the Netherlands. BMC Surg.

[5] Sheridan RL, Schomaker KT, Lucchina LC, Hurley J, Yin LM, Tompkins RG (1995). Burn depth estimation by use of indocyanine green fluorescence: Initial human trial. J Burn Care Rehabil.

[6] Still JM, Law EJ, Klavuhn KG, Island TC, Holtz JZ (2001). Diagnosis of burn depth using laser-induced indocyanine green fluorescence: A preliminary clinical trial. Burns.

[7] Fourman MS, Phillips BT, Crawford L, McClain SA, Lin F, Thode HC (2014). Indocyanine green dye angiography accurately predicts survival in the zone of ischemia in a burn comb model. Burns.

